# Thin Film Coating with Highly Dispersible Barium Titanate-Polyvinylpyrrolidone Nanoparticles

**DOI:** 10.3390/ma11050712

**Published:** 2018-05-01

**Authors:** Jinhui Li, Koji Inukai, Yosuke Takahashi, Akihiro Tsuruta, Woosuck Shin

**Affiliations:** 1 Department of Frontier Materials, Nagoya Institute of Technology, Nagoya 466-8555, Japan; kinki-ri@aist.go.jp; 2 Inorganic Functional Material Research Institute AIST, Nagoya 463-8560, Japan; a.tsuruta@aist.go.jp; 3 R&D Center, Noritake Co., Ltd., Miyoshi 470-02, Japan; koujiinukai@n.noritake.co.jp (K.I.); yosuke-takahashi@n.noritake.co.jp (Y.T.)

**Keywords:** BaTiO_3_, poly(vinylpyrrolidone), dispersion, bar coating, thin film, transmittance

## Abstract

Thin BaTiO_3_ (BT) coating layers are required in various multilayer ceramic technologies, and fine nanosized BT particles with good dispersion in solution are essential for this coating process. In this work, cubic and tetragonal phase monodispersed BT nanoparticles—which were referred to as LBT and HBT-PVP coated on their surface by polyvinylpyrrolidone (PVP) polymer—were prepared by low temperature synthesis (LTS) and hydrothermal method (HT) at 80 and 230 °C, respectively. They were applied for the thin film coating on polyethylene terephthalate (PET) and Si wafer substrates by a simple bar coating. The thickness of BT, LBT-PVP, and HBT-PVP films prepared by their 5 wt % coating agent on Si are around 268, 308, and 263 nm, and their surface roughness are 104.6, 91.6, and 56.1 nm, respectively. The optical transmittance of BT, LBT-PVP, and HBT-PVP films on PET are 55, 66, and 73% at 550 nm wavelength and the haze values are 34.89, 24.70, and 20.53% respectively. The mechanism of dispersant adsorbed on the BT surface for densification of thin film during the drying process of the film was discussed.

## 1. Introduction

BaTiO_3_ (BT) is widely used in multilayer ceramic capacitors, semiconductors, positive temperature coefficient thermistors, and piezoelectric devices [1,2] owing to its ferroelectric [3,4] and piezoelectric [5,6,7] properties. In recent years, its characteristic of wide band gap and high refractive index is also attractive [8,9,10]. High refractive index thin film can be used in waveguide sensors, optical filters, and anti-reflection coatings. Thin films of BT have a great potential application in erasable image storage devices and optical applications, such as non-linear optical systems, optical computing, and light guiding [11,12].

In recent years, with miniaturization of devices and thin films, the demand for nanosized BT has increased. Many processing methods for ferroelectric, high refractive index thin films have been developed for this purpose, such as chemical solution deposition by the sol–gel process [13] or physical depositions by sputtering [14], pulsed laser ablation [15], and chemical vapor deposition [16]. Each of these processes has advantages and disadvantages. Among these, the sol–gel coating method is the most popular process to obtain large-area thin films with good homogeneity and smooth surface at low temperatures. In the sol–gel process, films are deposited by dip coating [17,18] and spin coating [19,20], followed by high-temperature treatment.

As the particle size of BT decreases, the aggregation of nanoparticles becomes a serious problem. This induces defects in the thin films and imparts an uneven surface, and the nanoparticles cannot exhibit their inherent characteristics, which reduces the performance of the electrical components. In order to solve this problem, highly dispersible BT nanoparticles were prepared with polyvinylpyrrolidone (PVP) by the low temperatures synthesis (LTS) and hydrothermal (HT) process, referred to as LBT-PVP and HBT-PVP respectively, as reported in our previous paper [21,22]. The nanoparticles prepared by these methods can be dispersed as suspension in a coating slurry, and with these particles thin film layers can be prepared easily and quickly by a bar coating process as Itoh et al. reported [23]. This process does not require high-temperature treatment and the performance of the film depends on the crystalline phase of the powder. The characteristic of this process is that there is no troublesome process and the performance of the thin-film can be easily controlled. It is expected that highly dispersible nanomaterials contribute to the densification of the film and improve the performance of the device by this method.

In the present work, BT-PVP films of BT-PVP were prepared, and for comparison the film of commercial BT particle was also prepared. The characteristic of this process is that it neither involves complicated procedures nor high-temperature treatment, because the phase of the BT-PVP particles can be controlled by synthesis method. The performances of the thin film—such as morphology, thickness, and roughness—were evaluated in this study, which is expected to be related the dispersion of nanoparticle and surface of PVP, contributing to the dense and lower defect coating films and eventually improve the performance of the device. Optical transmittance in visible range of the thin films of different BT particles was also evaluated also. 

## 2. Results and Discussion

### 2.1. Properties of BT-PVPs Prepared by Different Synthesis Method

The crystalline phases of commercial BT and LBT-PVP prepared by LTS process are cubic, and that of HBT-PVP prepared by HT process is tetragonal as reported in our previous paper [21,22]. The tetragonality (c/a) of the particle is 1.0008, 1.0005, and 1.0058 for BT, LBT-PVP, and HBT-PVP respectively. The particle size and tetragonality (c/a) values of these particles in this study are list in Table 1 in detail.

**Figure 1:**
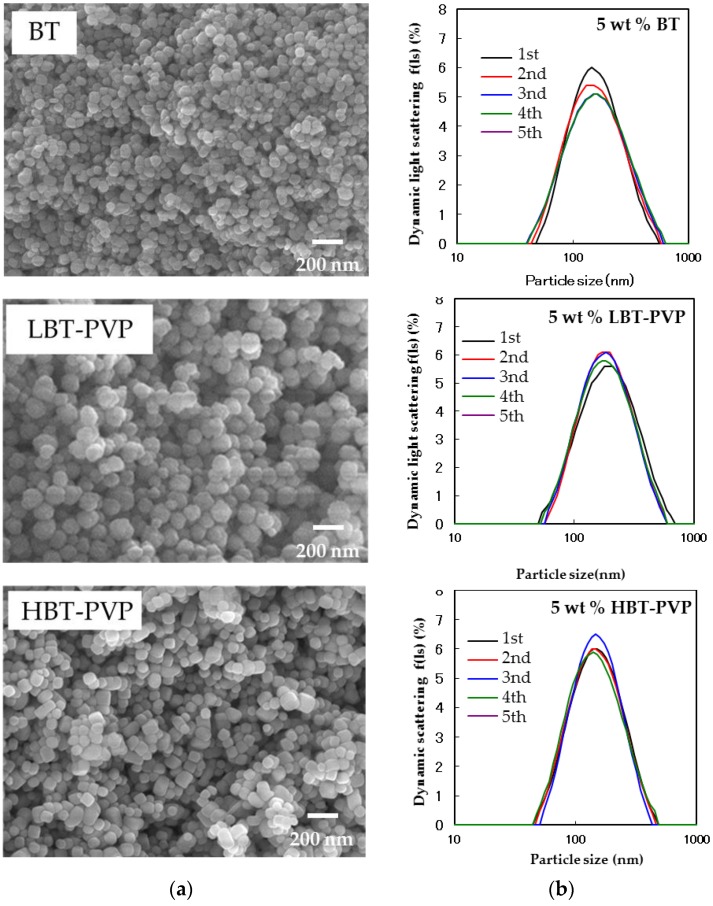
FE-SEM images (**a**) and dynamic light scattering (DLS) (**b**) of 5 wt % BT, LBT-PVP, and HBT-PVP in EtOH solution. The measurement of DLS is sequenced five times.

Figure 1 shows the FE-SEM images and dynamic light scattering of BT, LBT-PVP, and HBT-PVP, and their particle size measured by both SEM and DLS analysis. BT and LBT-PVP are spherical with a particle size of 81.0 nm (CV: 20.1%), and 128 nm (CV: 16.6%) as measured by FE-SEM. The particle sizes measured by DLS, are 141 nm (CV: 55.2%) and 164 nm (CV: 48.2%) respectively. The particle size of the dried powder evaluated by FE-SEM and the DLS particle size in EtOH slurry are so similar that BT and LBT-PVP can be considered monodisperse in EtOH. The cube-shaped HBT-PVP particles have a similar particle size of 106 nm (CV: 20.7%) and 131 nm (CV: 47.2%), as measured by FE-SEM and DLS, respectively, and demonstrate a monodisperse state in EtOH. Although the particle sizes of these three powders are not exactly the same, each of them is monodispered in EtOH solution.

**Figure 2:**
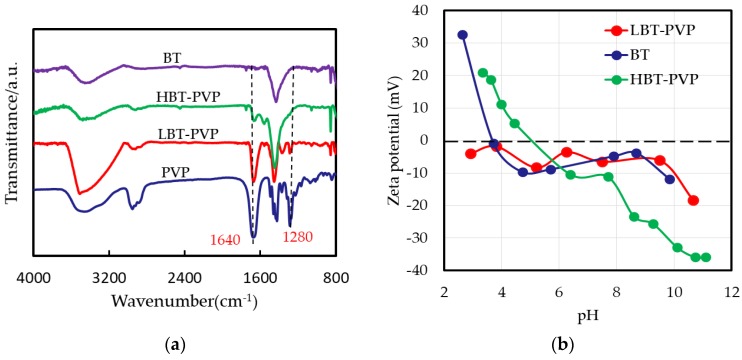
(**a**) FT-IR spectra of BT, HBT-PVP LBT-PVP and PVP; (**b**) relationship of zeta potential and pH of BT, LBT-PVP, and HBT-PVP in H_2_O.

The surface of BT, LBT‒PVP, and HBT‒PVP were characterized by FT-IR as shown in Figure 2a. The peak of 1640 cm^−1^ can be attributed to symmetric C=O stretching, and 1280 cm^−1^ corresponds to ring (CH_2_) wag +(CN) stretching of PVP rings [24]. It can be seen that the structure of dispersant of PVP does not change on the particle surface of LBT-PVP, but decomposes on the HBT-PVP, where the peak of 1280 cm^−1^ is not observed. Although the dispersant adsorbed on particle different, BT-PVPs demonstrate high dispersion.

It is well known that the zeta potential (ζ) depends on the pH value following Helmholtz–Smoluchowski formula:

(1) $$\zeta =\frac{\eta }{\varepsilon }\frac{v}{V}$$

where *η*, *υ*, *ε*, and *V* are solution viscosity, electrophoretic mobility, relative permittivity of solution, and voltage respectively. Because the pH effects the electrophoretic mobility of particle. The high dispersion of LBT-PVP depends on the steric effect of PVP for the (ζ) does not change with pH, and HBT-PVP depends on the electrostatic repulsion of decomposed PVP for the ζ changed with pH. The ζ of HBT-PVP is as low as −25 mV, as the pH of its suspension is 9.3 before the pH is adjusted, as shown in Figure 2b. In the other words, HBT-PVP is coated by negative-charged ionic dispersant as our previous reported results indicate [21,22]. On the contrary, the ζ of BT particles depends on pH, and the value is around 0–15 mV as pH is 4–10. It indicates that BT nanoparticles aggregate in aqueous solution in a wide pH range, although these results are obtained in aqueous solution, the dispersion mechanism is not different in EtOH. The ζ of the particle in EtOH would be large because the relative permittivity of EtOH (25.3) is smaller than water (80). The particles used in this study have high zeta potential in EtOH, leading them to demonstrate high dispersion in EtOH as shown in Figure 1b.

### 2.2. Properties of the BT-PVP Thin Film

**Figure 3:**
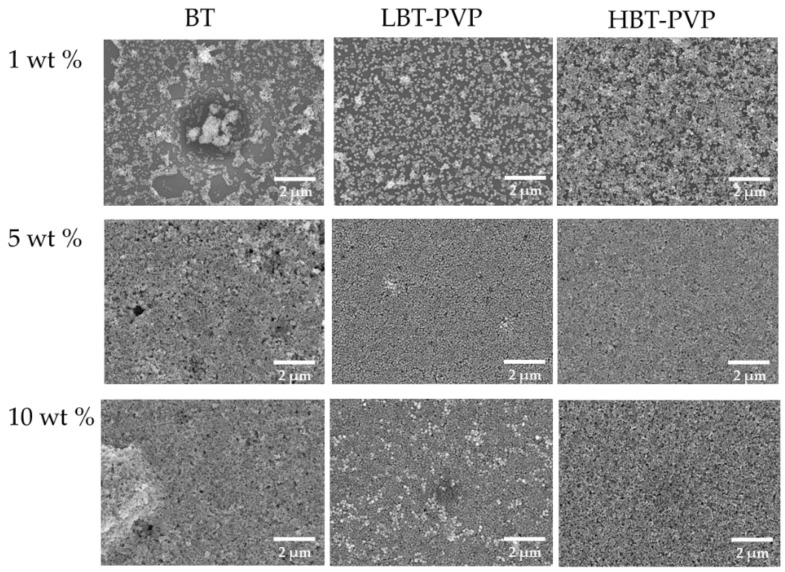
SEM images of the thin films prepared using different content of BT, LBT-PVP, and HBT-PVP in EtOH coated on PET. The contents of particles in EtOH are 1, 5, 10 wt %.

**Figure 4:**
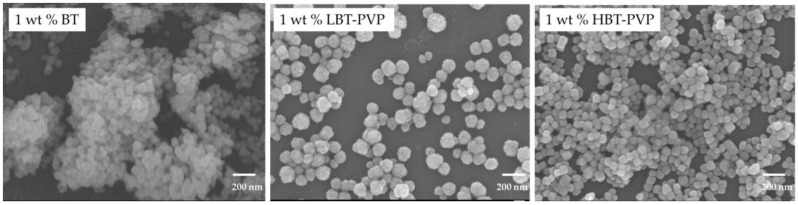
Enlarged SEM images of the thin films prepared 1 wt % BT, LBT-PVP, and HBT-PVP in EtOH coated on PET.

The high dispersion characteristics of BT, LBT-PVP, and HBT-PVP were used to prepare BT films on PET and Si substrates. Suspensions of BT, LBT-PVP, and HBT-PVP in EtOH with different contents were used as the coating agents. The film morphologies observed by FE-SEM, are shown in Figure 3. It can be seen that the density of the film filled by powder increases with the particle content increasing from 1 to 10 wt %. The role of dispersant coated on the particle surface can be also observed in 1 wt % film. Although each of them has high dispersion in EtOH, but morphologies of films are very different. There are large aggregates in BT films here and there for BT without dispersant coating, as shown in Figure 4. The particles of LBT-PVP are evenly dispersed on the PET substrate. It is thought that the dispersant PVP coated on the nanoparticle prevents the aggregates of nanoparticles during EtOH evaporation.

The HBT-PVP film looks relatively denser than LBT-PVP film, because uncoated PET areas of LBT-PVP film is more frequent as shown in Figure 4. This is due to the particle aggregations of BT films and LBT-PVP films as shown in Figure 3, and this low surface coverage is more serious in the case of BT film. Although all the DLS results of three different particles in EtOH are very good, their film formation or particle assembly during the drying process are different. It is speculated that both the steric effect of PVP and electrostatic repulsion are assisted with the capillary force which assemble the dispersed particles during the drying process, and a short-range assembly of nanoparticles is built in the case of HBT-PVP. In other words, the dispersant of decomposed PVP on BT-PVP is and effect not only for the dispersion of nanoparticles but also for close packing particle assembly, leading to dense and flat films.

The films of LBT-PVP and HBT-PVP made by 5 wt % are very dense and smooth, but the film made by BT have many holes and large particles aggregate. The role of dispersant, which prevents the aggregation of particles during evaporation of EtOH in the drying process of BT-PVP film, is also observed (Figure 3).

**Figure 5:**
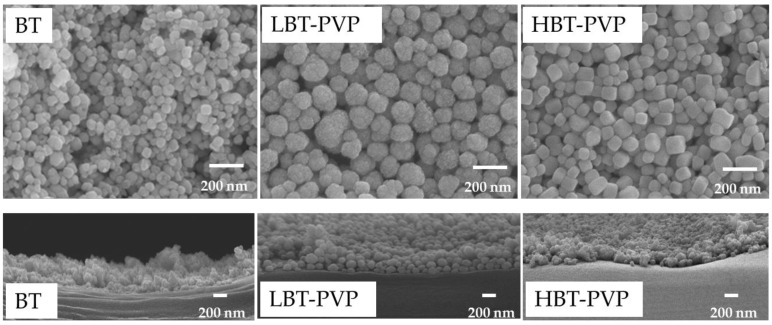
SEM images of the thin films surface (**top**), and cross section (**down**). These films were prepared by 5 wt % BT, LBT-PVP, and HBT-PVP in EtOH coated on PET.

Figure 5 shows the magnified SEM images of thin surface and their cross section that were prepared by 5 wt % coating agents on PET. It can be seen that the particles of BT-PVP are densely arranged in same height, but unevenness can be observed for the film of BT. It indicates that BT-PVP is easy to prepare smooth and dense thin film. The BT film has a very relatively rough surface, while the BT-PVP film exhibited a relatively uniform surface, with a thickness of around 200–300 nm measured from the cross-section images.

**Figure 6:**
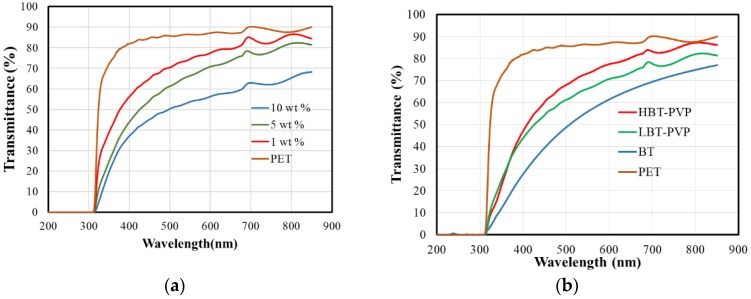
(**a**) Transmittance spectra of LBT-PVP films prepared in different concentration suspension from 1 to 10 wt % in EtOH. (**b**) Optical transmittance spectra of films prepared by different particles of BT, LBT-PVP, and HBT-PVP on PET. The particle content of coating agent is 5 wt %.

The coating of PVP on the surface of BT, which plays an important role in particle dispersion and uniform thin coating, is definitely a significant advantage in the industrial application of multilayer ceramics. Transparent oxide coating can be applicable to optoelectronics or UV-shield film, and the optical transmittance of BT films for UV–visible range is an important parameter. Figure 6a shows the transmittance spectra of LBT-PVP film prepared in different concentration suspension. Overall transmittance of LBT-PVP is lower than PET substrate. The 1 wt % film shows high transmittance for the particle coverage on the PET substrate is very low as shown in Figure 4. Transmittance of 5 and 10 wt % films decreases as the particle content increases. This decrease can be understood by the light scattering with thickness of film increase. 

In visible region (380–750 nm), the transmittance, BT, LBT-PVP, and HBT-PVP film is 55, 65, and 75% at 550 nm wavelength, respectively as shown in Figure 6b. Overall transmittance of BT-PVP films exceeds 60% in the visible region, and maximum transmittance of HBT-PVP film has reached to 80%. It indicates that it is advantageous the use of PVP coated on particle surface to prepare a film of high transmittance. The haze values of these films were also measured, and are 34.89, 24.70, and 20.53% for BT, LBT-PVP, and HBT-PVP film respectively (list in Table 2). Itoh et al. reported [25] that the transmittance of short wavelength visible light and haze value decrease with increasing nanoparticle size based on Rayleigh scattering theory. In this study, the particle size of BT is small (81 nm), that of HBT-PVP (106 nm) is medium, and that of LBT-PVP (128 nm) is large. However, the trend of transmittance and haze value of their thin film are different to the results obtained by Itoh et al. This leads us to the conclusion that BT nanoparticle have a strong aggregate and the aggregates as large particles scatter visible light, but BT-PVP have less aggregates on PET substrate and the particle size is relatively small. Maneehyya et al. [26] reported the sharp fall in the absorption edge at 330 nm for the BT film of sol–gel coating which is due to make a smooth surface of BT coating processed by liquid source. However, the broad absorption in this study is considered to be due to the surface roughness of BT particle coating, scattering the light waves.

**Figure 7:**
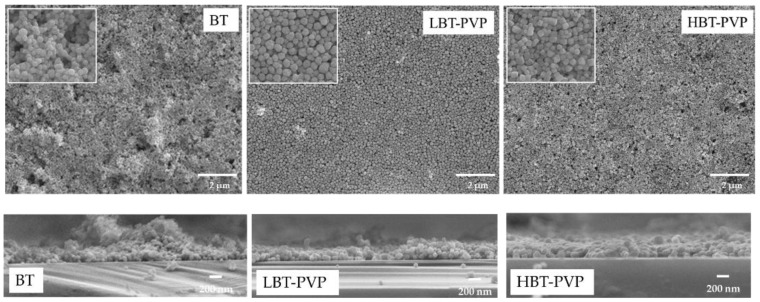
SEM images of film surface (**top**) and cross section (**down**). The film were prepared by 5 wt % BT, LBT-PVP, and HBT-PVP in EtOH coated on Si substrate. The insets are high-magnification SEM images of the films surface.

Presently, silicon wafers are extremely utilized substrates to build electronic devices, BT thin films on the Si substrates is expected as microelectronic devices [27]. In this study the film is also coated on Si substrate with 5 wt % coating agents. Figure 7 shows the surface and cross section morphology of the films coated on Si wafers. The same trend of morphology can be observed as on PET (Figure 3 and Figure 5). The surface of BT film is rougher compared to those of PVP-coated BT films. The particles are densely arranged in the films prepared by high-dispersion BT-PVP, but the particles aggregate in the film prepared by BT as shown in the high-magnification SEM images. The thickness of these films are estimated to be about 209, 292, and 293 nm from the cross section images for the BT, LBT-PVP, and HBT-PVP, respectively.

Another unique feature is that HBT-PVP films is a short-range face-to-face particle ordering of size 1 μm, regardless of the substrate (Figure 5 and Figure 7). This block of ordering can be formed during the evaporation of EtOH solvent, and considering both the thickness and roughness, it should be a plate-like two-dimensional assembly.

Recently, BT films composed of ordered cubic-shaped nanoparticles, so called nanocube assemblies, have been reported [18,19]. These state-of-the-art nanocube assembly films are fabricated by an evaporation-induced self-assembling process, resulting in a high degree of face-to-face ordering. However, this process requires a long time for the ordering of nanoparticles. The PVP-coated particles reported herein were well dispersed in the coating slurry and led to a short-range particle ordering in the film by a similar mechanism, but with much faster evaporation conditions.

**Figure 8:**
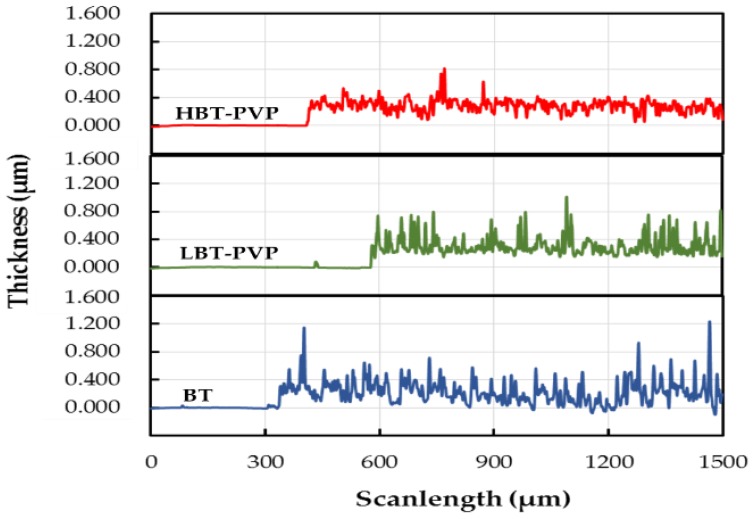
Stylus profiles of BT, LBT-PVP, and HBT-PVP films coated on Si substrates measured by stylus profiler.

Figure 8 shows the surface profiles of the BT, LBT-PVP, and HBT-PVP films coated on Si substrates. The thickness and roughness of the BT, LBT-PVP, and HBT-PVP films coated on Si substrates are listed in Table 3. The average surface roughness of the BT, LBT-PVP, and HBT-PVP films are 105, 91.6, and 56 nm, while the thicknesses (on Si substrate) are 226, 308, and 283 nm, respectively (list in Table 3), which are close to the values measured by FE-SEM (209, 292, and 293 nm, respectively). The roughness of LBT-PVP is higher than that of HBT-PVP. It demonstrates that the dispersant that decomposed PVP coated on HBT-PVP particle is more effective in preventing the agglomeration of particles during evaporating of EtOH than that PVP adsorbed on LBT-PVP on Si substrate. In other words, the electrostatic repulsion of decomposed PVP is more effective to prepare the smooth film than the steric effect of PVP.

It is extremely easy to coat the particle film on the substrates with the slurries prepared from BT-PVP particles in a uniform way. The thickness of the film can be controlled by choosing the wire diameter of the bar. As explained in the method, a single stroke of coating roughly deposits a 0.025 mm thick wet slurry film (from the diameter of winding of 0.5 mm and geometrical calculation). An extremely low particle concentration, such as 1 wt %, can lead to a low surface coverage; however, a defect-free clean layer can be prepared from 5 wt % particle content. Considering the particle content of 5 wt % and density of 6.02 g/cm^3^, the volume ratio of ~1 vol % corresponds to a dried film thickness of 250 nm. This estimation corresponds well to the thickness of the observed layer, which is also confirmed from the cross-sectional morphology shown in Figure 7 (data listed in Table 3).

Deposition of smooth and defect-free thin BT films by the sol–gel method has been previously reported [13], and films with thicknesses less than 1 μm can be prepared easily. However, it is necessary to crystallize the film by heat treatment in high temperature. Nevertheless, BT film is limited to the cubic phase, which does not show dielectric properties and requires a thermal post-treatment. Depending on the process, the sol–gel-deposited films remain in the cubic phase even after heat treatment at 1100 °C [20]. For thick films with above several microns in thickness, films are coated by the screen printing process using BT particles from solid-phase reaction; however, they yield much thicker and coarser films with many defects [28].

It is noteworthy that the coated films can be controlled in a narrow range of 0.1–0.3 μm thickness of the nanoparticles by a single stroke of bar coating in our study. Furthermore, the performance of the film can be controlled easily during the particle synthesis step. Considering industrial applications, the coating of the BT-PVP film on a PET substrate demonstrated in this report has the significant advantage of room-temperature coating without thermal treatment.

## 3. Materials and Methods

Titanium tetrachloride (TiCl_4_, 90+) and barium chloride dehydrate (BaCl_2_·2H_2_O, 90%) precursors as well as potassium hydroxide (KOH, 85%) mineral agent were purchased from Wako Pure Chemical Industries Ltd., Tokyo, Japan. The dispersant, PVP, with a molecular weight of 10,000 g/mol was obtained from Sigma-Aldrich Corp. (St. Louis, MI, USA).

BT particles coated by PVP were prepared following the procedure reported in our previous reports [21,22] by the LTS and HT methods. In the LTS process, the raw material of 0.2 M TiCl_4_ and BaCl_2_, 1.8 M KOH, and 100 g/L PVP were mixed in a vessel, and reacted at 80 °C for 1 h. In the HT process the precursors of 0.2 M TiCl_4_ and 0.32 M BaCl_2_, 2.3 M KOH, and 100 g/L PVP were mixed in a 50-mL Teflon container mixed with 20 mL of distilled water (75 vol %) and ethanol (25 vol %), and heated in the autoclave to 230 °C for 24 h. After completion of reaction, the solution was centrifuged to obtain the precipitates, which were washed and dried at 60 °C for 24 h. For comparison, commercial BaTiO_3_ (BT) powder (Sigma-Aldrich Corp., St. Louis, MI, USA) was used in this study. 

BT and BT-PVPs EtOH suspensions were used as coating agents. The coatings were applied to polyethylene terephthalate (PET; Toray, Tokyo, Japan) and Si substrates using a bar coater (Matsuo Sangyo Co. LTD., Osaka, Japan) with a coating speed of about 1.3 m/min. The standard bar RSD20 which its diameter is 6 mm and diameter of winding is 0.5 mm was used to control the thickness of the films. After coating, the films were dried in ambient atmosphere for 5 min and then at 80 °C for 8 min. 

The powder was characterized by X-ray diffraction (XRD; Smartlab, Rigaku Corp., Tokyo, Japan) using a standard diffractometer with CuKα radiation as an X-ray source in the 2*θ-θ* scan mode. In order to evaluate the tetragonality of the BT particles, the peak corresponding to the (200) plane was scanned at 0.01°/min in the range of 44–46° and fitted by the Gaussian function to deconvolute into two tetragonal peaks corresponding to (200) and (002) planes; the *c* and *a* lattice parameters were calculated as reported by Kwon et al. [29]. The morphologies of the films were observed by field-emission scanning electron microscopy (FE-SEM: JSM-6335FM, JEOL Ltd., Tokyo, Japan ) In addition, the size distribution of the particles was determined from the FE-SEM images, and the film thickness was measured from the cross-section FE-SEM images. The chemical structure of PVP adsorbed on the BT were evaluated by Fourier transform infrared spectroscopy (FT/IR-610, JASCO Corp., Tokyo, Japan). 

Dynamic light scattering (DLS; FPAR-10001, Otsuka Electronics Co., Ltd., Osaka, Japan) was used to evaluate the dispersibility and size distribution of the particles in an EtOH solution—a coating slurry containing 1, 5, and 10 wt % powder, respectively. Five sequential DLS measurements were made for each data point. The variations in particle size and distribution measured by SEM and DLS were evaluated by a coefficient variation (CV) method. The surface roughness of the film was measured using a surface profilometer (P-17 stylus profiler, KLA-Tencor Co., Milpitas, CA, USA), which includes the evaluation of film thickness. Transmittance of light by BT-PVP film on PET were characterized using JASCO V-670 ultraviolet–visible (UV–vis) spectrophotometer. The haze value was evaluated by a Nippon Denshoku Industries NDH 5000 haze meter, equipped with a white light emitting diode as the light source. Zeta potentials (ξ) of the synthesized BT–PVP and BT nanoparticles in aqueous solution were measured by electrophoretic light scattering (ELS–Z1/Z2, Ostuka Electronics Co. Ltd., Osaka, Japan).

## 4. Conclusions

Monodispersed cubic and tetragonal BaTiO_3_ (BT) particles coated on its surface by PVP polymer was synthesized by low temperature directly synthesis and hydrothermal method respectively, and was applied for the thin film coating on PET and Si wafer substrates by a simple bar coating and drying process.

While the surface of BT film of commercial nanoparticle is coarse and has many defects due to the particle aggregation, smooth and dense film deposition of cubic LBT-PVP and tetragonal HBT-PVP particle with c/a = 1.0005 and 1.0058. The crystalline phase can be simply controlled during the synthesis process. Heat treatment is unnecessary after the film prepared with BT-PVP. The thicknesses of LBT-PVP and HBT-PVP films on Si substrate coated by a single stroke of bar coating process were 308 and 283 nm, and their surface roughnesses were 91.6 and 56.1 nm respectively. The coating of PVP on the surface of BT particle facilitated the formation of ultra-thin film via two-dimensional assembly of particles with thickness corresponding to 2–3 particle stacking. The mechanism of dispersant adsorbed on BT surface for densification of the thin film during the drying process of film is discussed. 

The optical properties of BT thin film is expected to be transparent in the visible range for the application of optoelectronic devices. The optical transmittances of BT, LBT-PVP, and HBT-PVP films at 550 nm wavelength are 55, 66, and 73%, respectively, and the fall in the absorption edge of LBT-PVP and HB-PVP film is not sharp in our study.

The thin coating film using BT-PVP particle in this work could be a potential promising candidate for the ultra-thin multilayer ceramic application, and would be advantageous in the powder electronics if the post-annealing temperature is limited in the process. The high transmittance of BT-PVP film is expected to be used in various electro-optics.

## Figures and Tables

**Table 1:** Comparison of BT, LBT-PVP, and HBT-PVP particles size and tetragonality. Average particle size was measured by SEM and Dynamic light scattering (DLS) using ethanol (EtOH) suspensions with 5 wt % particles.

Particles	Particle Size (nm)				Tetragonality (c/a)
	SEM	CV* (%)	DLS	CV* (%)	
BT	81.0	20.1	141	55.2	1.0008
LBT-PVP	128	16.6	164	48.2	1.0005
HBT-PVP	106	20.7	131	47.2	1.0058

CV*: coefficient of variation.

**Table 2:** Properties of BT, LBT-PVP, and HBT-PVP thin films coated on PET substrates. Particle concentration in the EtOH coating slurry is 5 wt %.

Particles	Thickness (nm)	Transmittance * (%)	Haze (%)
BT	268.2	55.91	34.89
LBT-PVP	307.9	66.33	24.70
HBT-PVP	266.8	73.31	20.53

* Transmittance is at 550 nm wavelength.

**Table 3:** Properties of BT, LBT-PVP, and HBT-PVP thin films coated on Si substrates. Particle concentration in the EtOH coating slurry was 5 wt % (Ra*: average roughness).

Particles	Film Thickness (nm)		Ra* (nm)
	SEM	Profiler	
BT	209.4	226.2	104.6
LBT-PVP	292.1	308.4	91.6
HBT-PVP	293.2	283.1	56.1
